# Epidemiology and genetic diversity of bovine leukemia virus

**DOI:** 10.1186/s12985-017-0876-4

**Published:** 2017-11-02

**Authors:** Meripet Polat, Shin-nosuke Takeshima, Yoko Aida

**Affiliations:** 1 Viral Infectious Diseases Unit, RIKEN, 2-1 Hirosawa, Wako, Saitama, 351-0198 Japan; 2 Nano Medical Engineering Laboratory, RIKEN, 2-1 Hirosawa, Wako, Saitama, 351-0198 Japan; 3 Bovine Leukemia Virus Vaccine Laboratory RIKEN, 2-1 Hirosawa, Wako, Saitama, 351-0198 Japan

**Keywords:** Bovine leukemia virus (BLV), BLV diagnostic approches, BLV genotyping methods, BLV epidemiology

## Abstract

Bovine leukemia virus (BLV), an oncogenic member of the *Deltaretrovirus* genus, is closely related to human T-cell leukemia virus (HTLV-I and II). BLV infects cattle worldwide and causes important economic losses. In this review, we provide a summary of available information about commonly used diagnostic approaches for the detection of BLV infection, including both serological and viral genome-based methods. We also outline genotyping methods used for the phylogenetic analysis of BLV, including PCR restriction length polymorphism and modern DNA sequencing-based methods. In addition, detailed epidemiological information on the prevalence of BLV in cattle worldwide is presented. Finally, we summarize the various BLV genotypes identified by the phylogenetic analyses of the whole genome and *env* gp51 sequences of BLV strains in different countries and discuss the distribution of BLV genotypes worldwide.

## Background

Bovine leukemia virus (BLV) is a retrovirus, an oncogenic member of the *Deltaretrovirus* genus, and the causative agent of enzootic bovine leukosis (EBL) [1, 2]. The *Deltaretrovirus* genus also includes human T-cell lymphotropic virus types I and II (HTLV-I and -II) and simian T-cell lymphotropic virus (STLV) [3, 4]. EBL is a contagious lymphoproliferative disease of cattle, characterized by B-cell lymphosarcoma, which occurs throughout the world [2, 5]. Although BLV can infect various immune cell populations, including CD5^+^ IgM^+^ and CD5^−^ IgM^+^ B-cells; CD2^+^, CD3^+^, CD4^+^, CD8^+^, and γ/δ T-cells; monocytes; and granulocytes in peripheral blood and lymphoid tissues of cattle [6–11], BLV-induced tumors usually arise from the CD5^+^ IgM^+^ B-cell subpopulation [12].

BLV infection can result in a variety of clinical outcomes [2]. The majority of BLV-infected cattle are asymptomatic carriers of the virus, neither showing any clinical signs nor any changes in lymphocyte count; however, a recent study showed that although lymphocyte counts were not elevated in BLV-infected but clinically normal cattle, CD5^+^ IgM^+^ B-cells were increased [11], and there is substantial evidence suggesting that BLV-infected but clinically normal cattle may exhibit a degree of immunological dysregulation leading to economic losses for various reasons including reduced milk production [13], a high incidence of infectious disease [14], and reproductive inefficiency [15]. Approximately one-third of infected cattle develop a benign form of non-malignant proliferation of untransformed B-lymphocytes, termed persistent lymphocytosis (PL). PL is typically characterized by a permanent and stable increase in the number of CD5^+^ IgM^+^ B-cells circulating in the peripheral blood. Less than 5% of infected cattle develop malignant B-cell lymphoma originating from mono- or oligo-clonal accumulation of CD5^+^ IgM^+^ B-cells after a relatively long period of latency. This malignant form of B-cell lymphoma is predominantly detected in cattle over 4–5 years old [16]. Such malignancies induce disruption of the spleen and remarkable enlargement of the lymph nodes, which can be visible under the skin. BLV-induced neoplastic cells can penetrate into the abomasums, right auricle of the heart, intestine, kidney, lung, liver, and uterus. The clinical signs of BLV-induced tumors are varied and primarily involve digestive disturbance, weight loss, weakness, reduced milk production, loss of appetite, and enlarged lymph nodes [17].

### BLV genome structure

**Fig. 1:**
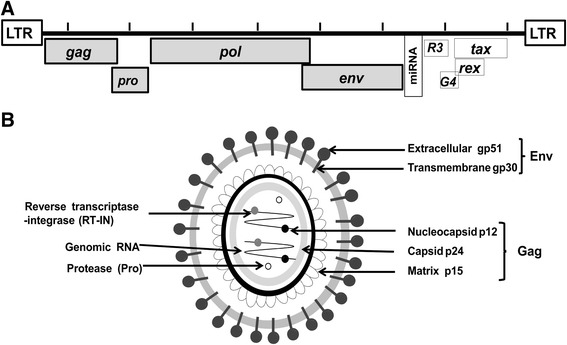
Schematic representations of the BLV genome structure (**a**) and viral particle (**b**). The structural and enzymatic genes, *gag*, *pro*, *pol*, and *env*; regulatory genes, *tax* and *rex*; accessory genes *R3* and *G4*; and microRNA (miRNA) are indicated in (**a**). Proteins encoded by structural and enzymatic genes, including the Env glycoproteins (gp51 and gp30) encoded by the *env* gene, the Gag proteins (p12, p24, and p15) encoded by the *gag* gene, reverse transcriptase and integrase (RT-IN) encoded by the *pol* gene, and protease (Pro) encoded by the *pro* gene are indicated in (**b**)

The BLV genome consists of 8714 nucleotides (nt) [18] including essential structural protein and enzyme coding genes and a *pX* region, flanked by two identical long terminal repeats (LTRs) (Fig. 1a). The structural protein and enzyme coding genes, namely, *gag*, *pro*, *pol*, and *env*, have essential and indispensable roles in the viral lifecycle, viral infectivity, and the production of infectious virions [19–24]. The *gag* gene of BLV is translated as the precursor, Pr45 Gag, and processed to generate three mature proteins [19, 23]: the matrix protein, p15, which binds viral genomic RNA and interacts with the lipid bilayer of the viral membrane [25]; the capsid protein, p24, which is the major target of the host immune response, with high antibody titers against this molecule found in the serum of infected animals [26, 27]; and the nucleocapsid protein, p12, which binds to packaged genomic RNA [28] (Fig. 1b). The *env* gene encodes the mature extracellular protein, gp51, and a transmembrane protein, gp30 [19]. The *pX* region, which is located between *env* and the 3′ LTR [2], encodes the regulatory proteins Tax and Rex, and the accessory proteins R3 and G4 (Fig. 1a). The regulatory proteins are important for regulation of viral transcription, transformation of BLV-induced leukemogenesis, and nuclear export of viral RNA into the cytoplasm [29–36]. The R3 and G4 accessory proteins contribute to the maintenance of high viral loads [37, 38]. In addition to the genes described above, the BLV genome also contains RNA polymerase-III-encoded viral microRNAs (miRNAs) between the *env* and *pX* regions. Viral miRNAs are strongly expressed in preleukemic and malignant cells, and may have roles in tumor onset and progression [39, 40] through their effects on proviral load and consequently viral replication in the natural host [41]. Besides, Van Driessche et al. revealed the recruitment of positive epigenetic marks on BLV miRNA cluster, inducing strong antisense promoter activity [42]. They also identified *cis*-acting elements of an RNAPII-dependent promoter [42].

### BLV diagnosis

A variety of techniques have been developed for diagnosis of BLV and implemented worldwide. These diagnostic methods can be assigned into two main groups, consisting of antibody-based serological tests and detection of the proviral genome by nucleic acid-based polymerase chain reaction (PCR) assays (summarized in Table 1).

**Table 1:** Summary of common techniques used for diagnosis of BLV prevalence

Diagnostic assay		Sample	Target	Advantages	Disadvantages	References
Type	Assay					
Serological test	AGID	Serum	Antibodies (p24, gp51)	Specific, simple, and easy to perform Large scale screening Less expensive Rapid	Less sensitive and inconclusive Cannot evaluate disease states of infected cattle	Aida et al., 1989 [47]
						Wang et al., 1991 [48]
						Monti et al., 2005 [49]
						Kurdi et al., 1999 [50]
						Jimba et al., 2012 [43]
						Naif et al., 1990 [55]
	ELISA	Serum Milk Bulk milk	Antibodies (p24, gp51)	Specific and sensitive Large scale screening Time saving	False negatives (cattle in early infection phase) False positive (maternally derived antibodies) Cannot evaluate disease states of infected cattle A number of controls and a plate reader required Results require interpretation	Naif et al., 1990 [55]
						Burridge et al., 1982 [56]
						Schoepf et al., 1997 [53]
						Kurdi et al., 1999 [50]
						Monti et al., 2005 [49]
						Jimba et al., 2012 [43]
						Zaghawa et al., 2002 [52]
	PHA	Virus particle	BLV glycoprotein	Sensitive Specific detection of BLV Large scale titration Less expensive Rapid	Affected by pH and temperature Hemagglutination activity reduced by trypsin, potassium periodate, and neuraminidase	Fukai et al., 1999 [51]
	RIA	Serum	Antibodies (p24)	Sensitive Able to detect BLV during the early period of infection	Cannot be used for mass screening	Levy et al., 1977 [54]
						Nguyen et al., 1993 [57]
Proviral DNA detection	Single PCR; Semi-nested PCR; Nested PCR	Blood PBMC Tumor sample Buffy coat Milk somatic cells Semen Saliva Nasal secretions	Provirus	Direct, fast, sensitive A variety of samples can be used BLV detection during the early phase of infection or in the presence of colostrum antibodies Can detect new infections, before the development of antibodies to BLV	Unable to detect BLV when the proviral load is too low Cross contamination occurs easily Requires specific primers Requires equipment (PCR machine) False negatives in the presence of PCR inhibitory substances in samples Requires internal control Needs confirmatory testing, such as sequencing	Monti et al., 2005 [49]
						Kurdi et al., 1999 [50]
						Zaghawa et al., 2002 [52]
						Tajima et al., 1998 [64]
						Tajima et al., 2003 [61]
	Real-time PCR	Blood PBMC Tumor sample Buffy coat Milk Somatic cells Semen Saliva Nasal secretions	Provirus	Direct, fast, sensitive Low risk of contamination A variety of samples can be used Distinguishes EBL from SBL BLV can be detected during the early phase of infection or in the presence of colostrum antibodies Quantitative measurement of proviral load	Requires internal control Requires positive controls of different concentrations Requires specific primers and probes Require equipment (real-time PCR machine) Expensive Complicated sample preparation procedure	Somura et al., 2014 [68]
						Lew et al., 2004 [69]
						Jimba et al., 2010 [70]
						Jimba et al., 2012 [43]
						Tawfeeq et al., 2013 [67]
						Brym et al., 2013 [66]
						Takeshima et al., 2015 [71]
	Direct blood-based PCR	Blood	Provirus	Cost-effective No need for DNA purification Low risk of contamination	Unable to detect BLV when the proviral load is too low Results in failure if there are mismatches between the PCR primers and BLV sequences Relatively low sensitivity	Nishimori et al., 2016 [72]
						Takeshima et al., 2016 [73]

*AGID* agar gel immunodiffusion, *BLV* bovine leukemia virus, *EBL* enzootic bovine leukosis, *ELISA* enzyme-linked immunosorbent assay, *PHA* passive hemagglutination assay, *RIA* radio immunoassay

#### Serological tests

For indirect BLV diagnostic methods, particularly antibody-based tests, antibodies recognizing the p24 capsid protein encoded by the *gag* gene and the extracellular gp51 protein encoded by *env-gp51* are targeted. This is because antibodies against these proteins are produced shortly after BLV infection, can be detected 2–3 weeks post-infection, and remain detectable for the life of the host animal [43]. In addition, the p24 capsid protein is a major target for host immune responses, inducing high antibody titers [44], and gp51 invokes the expression of massive amounts of specific antibodies in infected animals [24, 45, 46]. Therefore, antibodies against these proteins are targeted for BLV diagnostics using conventional serological techniques such as agar gel immunodiffusion (AGID) [43, 47–50], passive hemagglutination assay (PHA) [43, 51], enzyme-linked immunosorbent assay (ELISA) [43, 49, 50, 52, 53], and radio immunoassay (RIA) [54]. Most of these serological methods aim to detect antibodies in bovine serum and milk, and the supernatants of BLV-infected cell cultures. AGID is relatively inexpensive and can be used to screen many serum samples simultaneously; however, it is not sufficiently sensitive [55] and it is not suitable for analysis of milk samples. ELISA is a highly sensitive and easily implemented procedure, and can be used to analyze both serum and milk samples; however, it requires a number of controls and produces both false-negative result in serum samples from cattle in the early phase of infection [55] and false-positive results in calves that contain maternally-derived antibodies [56]. PHA aims to detect BLV glycoproteins, but, PHA test efficiency is sensitive to pH, temperature, and trypsin. RIA is suitable for diagnosing BLV soon after animals are exposed, but not suitable for the purpose of mass screening [57]. Overall, these antibody-based detection methods cannot be used to test calves less than 6 months old, due to the presence of maternal antibodies, which may trigger false-positive results [58].

#### Proviral DNA detection

BLV can integrate into dispersed sites within the host genome [59] and appears to be transcriptionally silent in vivo [60–62] and remain in cellular genomes, even in the absence of detectable BLV antibodies. Indeed, transcription of the BLV genome in fresh tumor or peripheral blood mononuclear cell samples from infected individuals is almost undetectable by conventional techniques [60, 63]. Interestingly, one copy of the full-length proviral genome can be detected in BLV-infected cattle throughout the course of the disease [64]. Another study also demonstrated that BLV-induced tumors and BLV-infected cells contain provirus, with approximately four copies of proviral DNA in each tumor [65]. Hence, in addition to the routine diagnosis of BLV infection using the conventional serological techniques described above, nucleic acid-based PCR methods can greatly accelerate the detection of BLV prevalence.

A variety of PCR methods, including standard PCR [49, 50], nested PCR [33, 52, 64], real-time quantitative PCR (qPCR) [43, 66–71], and direct blood-based PCR [72, 73], have been extensively applied worldwide for BLV detection (Table 1). A variety of genes in the BLV genome are targeted for detection of BLV infection prevalence by direct diagnostic PCR methods, including the LTR region [43, 70, 71, 73–77], and the *gag* [78], *pol* [69, 79, 80], *env* [55, 79], and *tax* [68, 79] genes.

Importantly, the BLV provirus copy number is generally very low compared with that of host genes therefore, the majority of PCR systems designed to detect BLV used a nested design [64, 74, 76]. These nested assays are extremely sensitive, but also obtain false-positive results due to DNA contamination. However, the method requires expensive real-time PCR machines and reagents and involves difficult sample preparation protocols. Recently, a novel blood-based PCR system that amplifies target DNA regions without a requirement for DNA isolation and purification was developed [72, 73]. The assay can detect BLV provirus with high specificity and at low cost, facilitating timely identification of BLV-infected cattle.

As discussed above, PCR-based genome screening methods for diagnosis of BLV broaden the range of samples that can be used, increase testing sensitivity, specificity, and efficiency, and are less time consuming. PCR also allows the detection of BLV infection in cattle several weeks before it is possible to detect antibodies [81]; however, PCR-based provirus screening involves complicated sample preparation processes, which can lead to false-positive results if cross contamination occurs. In addition, PCR-based BLV detection methods require specific laboratory facilities, including PCR machines, and the design of specific primers and probes is also necessary. The CoCoMo algorithm, is a method used to design degenerate primer sets that amplify all available sequences within a target region. Recently, the BLV-CoCoMo-qPCR assay was developed to measure the BLV proviral load with extremely high sensitivity and to amplify both known and novel BLV variants [43, 70, 71]. This assay enabled us to demonstrate that the proviral load correlates not only with BLV infection capacity but also with BLV disease progression [43, 82], and identification of risk factor associated with increased BLV proviral load in infected cattle [82, 83] and detection of BLV provirus in nasal secretion and saliva samples [84].

#### Other methods

In addition to the techniques described above, other BLV diagnostic approaches, including detection of viral proteins by western blotting [21, 31, 33, 85], a syncytium formation assay [85], and detection of BLV antigens by indirect immunofluorescent assay [47], have also been described.

### BLV genotyping and identification of ten distinct genotypes

Studies of BLV genotypes for phylogenetic and epidemiological analyses have primarily focused on the *env* gene, the *env gp51* gene in particular, because of its biological functions. The extracellular gp51 protein has key roles in the viral lifecycle and is indispensable for viral entry into host cells [20, 86]. In addition, because of the surface localization of the gp51 glycoprotein, it is also the target of neutralizing antibodies [87]. The conformational epitopes, F, G, and H, located in the N-terminal half of gp51, are important in syncytium formation and viral infectivity [87, 88]. Therefore, the *env gp51* sequence region is frequently used for BLV phylogenetic analysis.

Over the years, a number of methods have been applied for BLV genotyping, as summarized in Table 2. In the early days of BLV genotyping, researchers clustered or genotyped BLV strains from different geographical regions based on restriction fragment length polymorphisms (RFLP) of PCR-products, generated using various restriction enzymes [86, 89–96]. BLV clusters and genotypes were named after the geographical region of sample isolation, such as “Argentine type” or “Australian type”, or with reference to phylogenetic clustering (e.g., “cluster one”). A total of seven BLV clusters/genotypes were determined by PCR-RFLP [91]; however, PCR-RFLP genotyping studies were not consistent or comprehensive.

**Table 2:** Summary of BLV genotyping methods

Genotyping method	Amplified BLV region	Amplicon size (bp)	Enzymes	Phylogenetic approaches	Classification result	Reference
PCR-RFLP	Partial *env-gp51* region	444	*BamH*I, *Bgl*I, *Hae*III, *Bcl*I, *PvuI*I, *Dra*I, *Hind*III, *Hpa*II, *Stu*I, *Taq*I		7 groups: A, B, C, D, E, F, G	Fechner et al., 1997 [90]
						Licursi et al., 2002 [91]
						Asfaw et al., 2005 [95]
RFLP + sequencing	Partial *gp51* sequencing	400–444	*Bam*HI, *Bcl*I, *Pvu*II, *Gmb*H	NJ; MP; ML	RFLP-based type: Australian type, Argentine type, Belgium type, Japanese type; Sequence-based type: Argentine cluster, European cluster, Japan and German isolate cluster; groups I–IV; or genotypes 1–8	Monti et al., 2005 [49]
						Felmer et al., 2005 [93]
						Camargos et al., 2007 [122]
PCR-sequencing	Partial *gp51* sequencing	346–444		NJ; ML; BI	Japanese group, Argentine group, European group; or genotypes 1–8	Camargos et al., 2002 [121]
						Licursi et al., 2003 [92]
						Matsumura et al., 2011 [98]
						Rola-Luszczak et al., 2013 [99]
						Polat et al., 2015 [74]
						Ochirkhuu et al., 2016 [77]
						Polat et al., 2016 [75, 76]
	Sequencing of partial or full *gp51* gene sequences	444–903		NJ; ML; BI	Up to 10 BLV genotypes	Moratorio et al., 2010 [126]
						Balic et al., 2012 [97]
						Lee et al., 2015 [100]
						Lee et al., 2016 [101]
	Sequencing of *env* (full *gp51* and/or *gp30* genes)	up to 1548		NJ; ML; BI	Consensus cluster, US Californian cluster, European cluster, Costa Rican cluster; or genotypes 1–10	Zhao et al., 2007 [109]
						Rodriguez et al., 2009 [96]
						Yang et al., 2016 [131]
Full BLV genome sequencing	BLV complete genome	8714		ML	genotypes −1, −2, −4, −6, −9, and −10	Polat et al., 2016 [75, 76]

*BI* Bayesian inference, *BLV* bovine leukemia virus, *NJ* neighbor-joining, *ML* maximum-likelihood, *MP* maximum-parsimony, *RFLP* restriction fragment length polymorphism

**Fig. 2:**
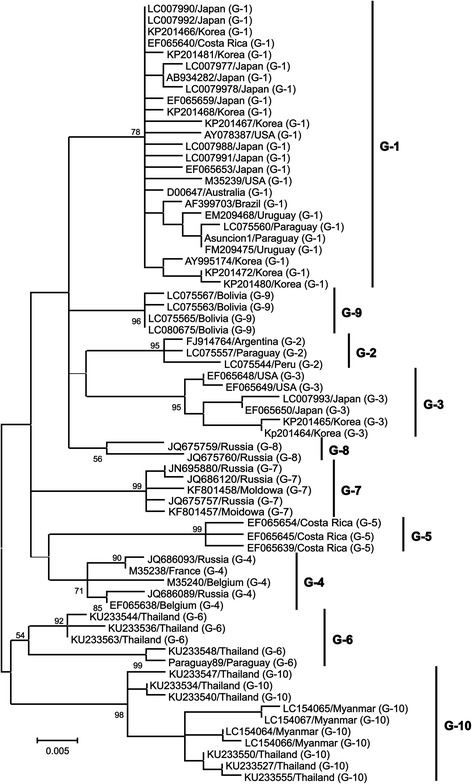
Maximum likelihood phylogenetic tree constructed based on partial BLV *env* sequences identified in geographical locations around the world. A maximum likelihood (ML) phylogenetic tree was constructed based on sequences from known BLV strains, representing ten different BLV genotypes derived from viruses isolated worldwide. Nucleotide sequences were obtained from the GenBank nucleotide sequence database. Sequences are labeled with their accession numbers and countries of origin. Genotypes are indicated by numbers to the right of the figure. One thousand replications were performed to calculate bootstrap values (indicated on the tree). The bar at the bottom of the figure indicates evolutionary distance

**Fig. 3:**
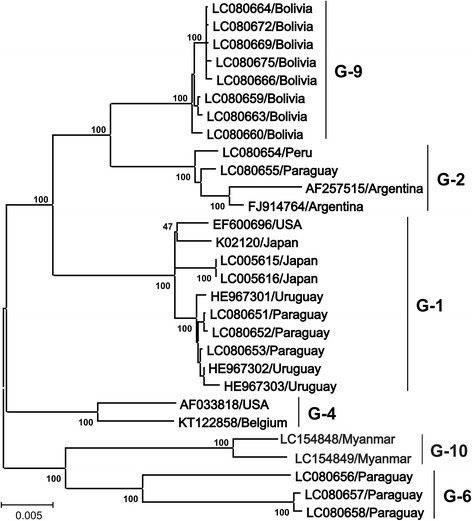
Maximum likelihood (ML) phylogenetic tree constructed from complete BLV genomic sequences. The ML phylogenetic tree was constructed using complete BLV genomic sequences from the GenBank nucleotide sequence database. One thousand replications were performed to calculate bootstrap values (indicated on the tree). The strains identified in this study are indicated by the sample identification number and country name. Genotypes are indicated by numbers to the right of the figure. The bar at the bottom of the figure indicates evolutionary distance

In 2007, Rodriguez et al. reported sequencing of the *env* gene (all of *gp51* and part of *gp30*) of 28 BLV field strains, performed phylogenetic analysis of these sequences in comparison with published sequence data representative of established genetic groups by neighbor-joining, maximum likelihood, and Bayesian inference methods, and assigned BLV sequences into seven genotypes [97]. Subsequently, a new genotype, genotype-8, was identified in BLV samples from Croatia by Balic et al. [98], who concluded that BLV may be more divergent than previously thought, speculating that additional genotypes might be discovered in the future. Indeed, the presence of eight BLV genotypes was later confirmed in different geographical locations [74, 77, 99–101]. Finally, in 2016, the novel BLV genotypes, genotype-9 and -10, were discovered in Bolivia [75], Thailand [102], and Myanmar [76], a totaling ten BLV genotype clusters (Fig. 2). Previously, almost all phylogenetic studies of BLV genotypes focused on the partial or entire *env* gene. However, for the first time in their study [75, 76], Polat et al. successfully concluded the existence of genotypes-1, −2, −4, −6, −9 and −10 among ten BLV genotypes (Fig. 3) by phylogenetic analysis using complete sequences of BLV strains newly determined by next generation sequencing and sequencing cloned, overlapping PCR products in their studies, and using complete BLV genome sequences available in the database (NCBI & DDBJ). These phylogenetic analysis of complete BLV genomes demonstrated that each BLV genotype encodes specific amino acid substitutions in both structural and non-structural gene regions.

### BLV prevalence

BLV has spread to all continents via the trade in breeding animals, and is prevalent in cattle worldwide. BLV infection levels vary between and within countries, as shown in Table 3 (data obtained on March 17th, 2017; updated and detailed information is available at http://www.oie.int/wahis_2/public/wahid.php/Diseaseinformation/statuslist) [17, 103]. BLV eradication programs and control measures have been established in European Community member countries since the second half of the twentieth century, and eradication programs have been very successful in the majority of western Europe [104–107]; indeed, some countries, including Denmark, Finland, Switzerland, Estonia, The Netherlands and Poland, are completely free of BLV [104, 108–110]. Despite the majority of countries in Western Europe being free from disease, EBL still exists in eastern European nations, including Poland, Ukraine, and Croatia [98, 100, 111–113]. In addition, in Italy, Portugal, Belarus, Latvia, Greece, Romania, and Bulgaria, BLV is present, although disease is either absent or limited to specific areas [103].

**Table 3:** Detailed information on BLV infection levels worldwide

Geographical division	Country	Within country	BLV prevalence^a^	References
Europe	Andorra	Nationwide	BLV-free, 1994	OIE, 2009 [103]
	Cyprus	Nationwide	BLV-free, 1995	OIE, 2009 [103]
	Czech Republic	Nationwide	BLV-free, 2010	OIE, 2009 [103]
	Denmark	Nationwide	BLV-free, 1990	OIE, 2009 [103]
	Estonia	Nationwide	BLV-free, 2013	OIE, 2009 [103]
	Finland	Nationwide	BLV-free, 2008	OIE, 2009 [103]
	Ireland	Nationwide	BLV-free, 1999	OIE, 2009 [103]
	Norway	Nationwide	BLV-free, 2002	OIE, 2009 [103]
	Spain	Nationwide	BLV-free, 1994	OIE, 2009 [103]
	Switzerland	Nationwide	BLV-free, 2005	OIE, 2009 [103]
	Sweden	Nationwide	BLV-free, 2007	OIE, 2009 [103]
	Slovenia	Nationwide	BLV-free, 2006	OIE, 2009 [103]
	UK	Nationwide	BLV-free, 1996	OIE, 2009 [103]
	The Netherlands	Nationwide	BLV-free, 2009	OIE, 2012 [17]
	Poland		BLV-free, 2017	EFSA Panel on Animal Health and Welfare, 2017 [110]
	Ukraine		Present	OIE, 2012 [17]; Rola-Luszczak et al., 2013 [100]
	Croatia		Present	OIE, 2012 [17]; Balik et al., 2012
	Italy		Present	OIE, 2009 [103]; Molteni et al., 1996 [144]
	Portugal		Present	OIE, 2009 [103]
	Belarus		Present	OIE, 2012 [17]; Rola-Luszczak et al., 2013 [100]
	Latvia		Present	OIE, 2009 [103]
	Romania		Restricted to certain area	OIE, 2009 [103]
	Bulgaria		Present	OIE, 2009 [103]
	Greece		Present	OIE, 2009 [103]
Oceania	Australia		BLV-free in dairy cattle, 2013	EPAHW, 2015 [113]
	New Zealand		BLV-free, 2008	Chethanond, 1999 [114]
North America	USA		83.9% dairy cattle; 39% beef cattle, 2007	APHIS, 2008 [115]
	Canada	Nationwide	89% at herd level	APHIS, 2008 [115]
		Nationwide	78% at herd level, 1998–2003	Nekouei, 2015 [13]
		Saskatchewan	37.2% at individual level, 2001	VanLeeuwen et al., 2001 [116]
		Maritime	20.8% at individual and 70.0% at herd level, 1998–1999	VanLeeuwen et al., 2005 [117]
		Maritime	30.4% at individual and 90.8% at herd level, 2013	Nekouei, 2015 [118]
	Mexico	Nationwide	36.1% of dairy and 4.0% of beef cattle, 1983	Suzan et al., 1983 [119]
South America	Brazil		17.1% to 60.8%, 1980–1989 and 1992–1995	Sammara et al., 1997 [120] ; D’Angelino et al., 1998 [121]
	Argentina	Buenos Aires	77.4% at individual and 90.9% at herd level, 2007	Polat et al., 2016 [75]
		Multiple regions	32.85% at individual and 84% at herd level, 1998–1999	Trono et al., 2001 [124]
	Chile	Southern region	27.9% at individual level, 2009	Polat et al., 2016 [75]
	Bolivia	Multiple regions	30.7% at individual level, 2008	Polat et al., 2016 [75]
	Peru	Multiple regions	42.3% at individual level, 2008	Polat et al., 2016 [75]
		Multiple regions	31.0% at individual level, 1983	Ch, 1983 [125]
	Venezuela	Nationwide	33.3% at individual level, 1978	Marin et al., 1978 [126]
	Uruguay		Present	Moratorio et al., 2010 [127]
	Paraguay	Asuncion	54.7% at individual level, 2008	Polat et al., 2016 [75]
	Colombia	Narino	19.8% at individual level, 2013	Benavides et al., 2013 [131]
Africa	South Africa		BLV-free, 2012	OIE, 2012 [17]
	Tunisia		BLV-free, 2005	OIE, 2009 [103]
	Egypt		BLV-free, 1997	OIE, 2009 [103]
Asia	Kazakhstan		BLV-free, 2007	OIE, 2009 [103]
	Kyrgyzstan		BLV-free, 2008	OIE, 2009 [103]
	China		49.1% of dairy and 1.6% of beef cattle, 2013–2014	Yang et al., 2016 [132]
	Japan	Nationwide	40.9% of dairy and 28.7% of beef cattle, 2009–2011	Murakami et al., 2013 [136]
		Nationwide	79.1% of dairy herd, 2007	Kobayashi et al., 2010 [134]
		Nationwide	28.6% overall; 34.7% of dairy, 16.3% of beef, and 7.9% of fattening beef cattle, 2007	Murakami et al., 2011 [135]
		Nationwide	73.3% at individual cattle, 2012–2014	Ohno et al., 2015 [83]
	Mongolia		3.9% of dairy cattle, 2014	Ochirkhuu et al., 2016 [77]
	Cambodia		5.3% of draught cattle, 2000	Meas et al., 2000 [137]
	Taiwan		5.8% of dairy cattle, 1986	Wang et al., 1991 [48]
	Iran	Nationwide	Between 22.1% to 25.4%, 2012–2014	Nekoei et al., 2015 [138]; Mousavi et al., 2014 [139].
		Khorasan Razavi	29.8% of dairy cattle, 2009	Mousavi et al., 2014 [139].
		Khorasan Shomali	1.5% of dairy cattle, 2009	Mousavi et al., 2014 [139].
	Thailand		58.7% of cattle, 2013–2014	Lee et al., 2016 [102]
	Philippines		4.8% to 9.7% of cattle, 2010–2012	Polat et al., 2015 [74]
	Myanmar		9.1% at individual level 2016	Polat et al., 2016 [76]
	Korea		54.2% of dairy cattle and 86.8% of dairy herds; 0.14% of beef cattle, 2014	Lee et al., 2015 [101]
Middle East	Israeli		5% at individual level	Trainin & Brenner, 2005 [140]
	Saudi Arabia		20.2% of dairy cattle, 1990	Hafez et al., 1990 [141]
	Turkey		48.3% of dairy herd	Burgu et al., 2005 [142]

BLV prevalence in this table shows BLV infection in certain specific period. Therefore, there might be a change in BLV prevalence in different times

*APHIS* Animal and Plant Health Inspection Service, *BLV* bovine leukemia virus, EFSA European Food Safety Authority, *EPAHW* European Panal on Animal Health and Welfare, *OIE* The World Organisation for Animal Health

**Note:** ^a^BLV prevalence in each sample collection year; however, no information about sample collection year was provided in some cases

Nationwide BLV eradication and control programs were introduced in Australia and New Zealand in 1983 and 1996, respectively, and 99.7% of Australian dairy herds were declared free from EBL in December 2013, while those in New Zealand have been free from BLV-induced EBL since 2008 [113, 114].

In North America, an epidemiological study of BLV prevalence in US dairy cattle conducted by the Department of Agriculture’s National Animal Health Monitoring System demonstrated that 83.9% of dairy cattle were BLV-positive at herd level and 39% of beef herds had at least one BLV-infected animal [115]. In Canada, studies of BLV prevalence revealed that up to 37.2% of cows and 89% of herds were BLV-positive [116–118]. BLV is also present in both beef and dairy cattle in Mexico [119]; however, disease is either absent or limited to specific areas [17] (accessed on 22 Dec 2016).

In South America, relatively high levels of BLV prevalence have been observed, and BLV-induced leukosis is present in the majority of countries. In Brazil, BLV prevalence varies among states, with infection rates ranging from 17.1% to 60.8% [120–123]. Individual and herd level BLV prevalence in Argentina are as high as 77.4% and 90.9%, respectively [75, 95, 124]. Moreover, individual infection rates between 19.8% and 54.7% have been reported in Chile, Bolivia, Peru, Venezuela, Uruguay, Paraguay, and Columbia [75, 94, 125–131].

BLV infection is widespread in Chinese dairy farms. Infection rates are up to 49.1% among individual dairy cattle, while 1.6% of beef cattle are BLV-positive [132]. Moreover, serological tests revealed that 20.1% of yaks in China were BLV-positive [133]. Epidemiological studies in Japan revealed varying levels of BLV prevalence throughout the country, based on different detection methods [83, 134–136], and BLV infection rates of 40.9% of dairy and 28.7% of beef cattle, with infection rates in animals over 2-years-old reaching 78% in dairy herds and 69% in beef cattle herds [136]. Less than 6% of cattle were infected with BLV in Mongolia (3.9%) [77], Cambodia (5.3%) [137], and Taiwan (5.8%) [48], while a serological survey in Iran revealed that the prevalence of BLV was between 22.1% and 25.4% in that country [138, 139]. Lee et al. [102] demonstrated an average prevalence of BLV of 58.7% in Thailand, reaching maxima of 87.8% and 100% of cattle when assayed using PCR and ELISA, respectively. In Korea, 54.2% of dairy cattle and 86.8% of dairy herds were BLV-positive, whereas only 0.14% of beef cattle were infected with BLV [101]. BLV infection levels in The Philippines ranged from 4.8% to 9.7% [74] while it was 9.1% in Myanmar [76]. BLV infections in Middle Eastern countries are relatively low. The prevalence of BLV infection is approximately 5% in Israel [140], while in Saudi Arabia, 20.2% of dairy cattle tested as BLV-positive [141]. Compared to these countries, BLV infection rates in Turkey are higher, with 48.3% of dairy herds including sero-positive animals [142].

### Distribution of BLV genotypes worldwide

As mentioned above, phylogenetic analyses of whole genome (Fig. 3) and *env* gp51 sequences (Fig. 2) of BLV strain showed that BLV can be classified into ten genotypes. Three genotypes of BLV, namely genotype-1, genotype-4 and genotype-6, were mainly detected from across the world, as shown in Table 4. Genotype-1 is the most dominant genotype of BLV and is distributed across almost all continents, including Europe, America, Asia, and Australia. In particularly, genotype-1 spread to South and North America, and these continents still have a high prevalence of BLV infection. In addition, genotype-1 continues to spread worldwide, including Asian countries. The second most widely distributed genotype is genotype-4, which is primarily detected in Europe and some American countries. However, it is only found in Mongolia among Asian nations. Interestingly, although genotype-4 used to exist in Europe, it decreased because of BLV eradication in European countries. Genotype 6 may have come from South America and spread to South Asia by animal trading. Of the other genotypes, genotype-2 is restricted to South American countries and is only found in Japan among Asian nations, while genotype-8 is restricted to Europe. Genotypes-5 (in Brazil and Costa Rica) and −10 (in Thailand and Myanmar) are only observed in geographically proximal areas, where there may be an exchange of animals across national boundaries [76, 102]. By contrast, genotypes-7 is distributed across geographically dispersed regions [74, 77].

**Table 4:** Worldwide geographical distribution of the ten known BLV genotypes based on *env-gp51* sequences

Geographical division	Country	Genotype										Reference
		1	2	3	4	5	6	7	8	9		
Europe	Belarus				4							Rola-Luszczak et al., 2013 [99]
	Russia				4			7	8			Rola-Luszczak et al., 2013 [99]
	Ukraine				4			7	8			Rola-Luszczak et al., 2013 [99]
	Croatia								8			Balic et al., 2012 [97]
	Poland				4			7				Rola-Luszczak et al., 2013 [99]
	Belgium				4							Mamoun et al., 1990 [85]; Zhao & Buehring, 2007 [142]
	France			3	4							Mamoun et al., 1990 [85]
	Germany	1			4							Fechner et al., 1997 [90]
	Italy							7				Molteni et al., 1996 [143]
Australia	Australia	1										Coulston et al., 1990 [89]
America	USA	1		3	4							Derse et al., 1985 [144]; Mamoun et al., 1990 [85]; Zhao & Buehring, 2007 [142]
	Caribbean	1										Yang et al., 2016 [145]
	Costa Rica	1				5						Zhao & Buehring, 2007 [142]
	Argentina	1	2		4		6					Dube et al., 2000 [146]; Licursi et al., 2003 [92]; Monti et al., 2005 [94]; Dube et al., 2009 [147]; Rodriguez et al., 2009 [96]
	Brazil	1	2			5	6	7				Camargos et al., 2002 [121]; Camargos et al., 2007 [122]; Moratorio et al., 2010 [126]
	Chile				4			7				Felmer et al., 2005 [93]
	Bolivia	1	2				6			9		Polat et al., 2016 [75]
	Peru	1	2				6					Polat et al., 2016 [75]
	Paraguay	1	2				6					Polat et al., 2016 [75]
	Uruguay	1										Moratorio et al., 2010 [126]
Asia	Korea	1		3								Lim et al., 2009 [148]; Lee et al., 2015 [100]
	Japan	1	2	3								Licursi et al., 2003 [92]; Zhao & Buehring, 2007 [142]; Matsumura et al., 2011 [98]; Inoue et al., 2011 [149]
	Philippines	1					6					Polat et al., 2015 [74]
	Thailand	1					6				10	Lee et al., 2016 [101]
	Myanmar										10	Polat et al., 2016 [76]
	Mongolia	1			4			7				Ochirkhuu et al., 2016 [77]
	Jordan	1					6					Ababneh et al., 2016 [150]

In detail, in Europe, a total of five different BLV genotypes have been detected (genotypes −1, −3, −4, −7, and −8): genotype-4 in Belarus [100] and Belgium [86, 143]; genotypes-4, −7, and −8 in Russia and Ukraine [100]; genotype-8 in Croatia [98]; genotypes −4 and −7 in Poland [100]; genotypes −3 and −4 in France [86]; genotypes −1 and −4 in Germany [91]; and genotype-7 in Italy [144]. In Australia, only genotype-1 was detected [90]. In North America, genotypes −1, −3, and −4 have been detected in the USA [86, 143, 145], and genotype-1 was reported in the Caribbean [146]. In Central America, genotypes −1 and −5 were detected in Costa Rica [143]. A variety of BLV genotypes (−1, −2, −4, −5, −7, and −9) were detected in South America: genotypes −1, −2, −4, and −6 in Argentina [93, 95, 97, 147, 148]; genotypes −1, −2, −5, −6, and −7 in Brazil [122, 123, 127]; genotypes −4 and −7 in Chile [94]; genotypes −1, −2, −6, and −9 in Bolivia [75]; genotypes −1, −2, and −6 in Peru and Paraguay [75]; and genotype-1 in Uruguay [126]. In Asia, a total of seven BLV genotypes have been confirmed (−1, −2, −3, −4, −6, −7, and −10): genotypes −1 and −3 in Korea [101, 149]; genotypes −1, −2, and −3 in Japan [93, 99, 143, 150]; genotypes −1 and −6 in The Philippines [74]; genotypes −1, −6, and −10 in Thailand [102]; genotypes −1, −4, and −7 in Mongolia [77]; genotype-10 in Myanmar [76]; and genotypes −1 and −6 in Jordan [151].

Based on the European Food Safety Authority panel on animal health and welfare, BLV-induced EBL may have originated and spread widely from an area of Memel in East Prussia (now Klaipeda in Lithuania) [113, 152]. The worldwide distribution of the disease occurred due to the introduction of cattle from European countries into herds in other countries free of the disease, and also through the international trade of bred animals [113]. Interestingly, genotype-4 existed primarily in East Prussia as shown in Table 4. Then, infected cattle were reintroduced into some European countries; for example, BLV was introduced into the UK via bred animals from Canada in 1968 and 1973 [113]. As detailed in some previous publications, the widespread distribution of BLV genotypes within and between distant geographical locations may be driven by the spread of virus through the movement of live animal populations, associated with human migration and animal domestication, and also with viral transmission during close contact between individual animals [97].

### Future prospects

It appears that at least ten different BLV genotypes of BLV strains are circulating in various geographical locations worldwide. The completion of whole genome sequencing of these BLV strains has revealed that BLV genomes contain a number of unique genotype specific substitutions not only in the *env* region, but also in the LTR, Gag, Pro, Pol, Tax, Rex, R3, G4, and miRNA encoding regions, distinguishing each genotype [75]. However, the BLV genome sequences of strains from different geographic origins, especially the important sites on the regulation of viral replication of BLV, are relatively stable and highly conserved among BLV strains, assigned to different genotypes. By contrast, several groups recently reported that the expression or pathogenesis of BLV does not depend on strains, but rather, is related with the specific site of mutation in their BLV genome [153, 154]. These results clearly demonstrate that BLV strain should be determined by full genome sequencing. However, although BLV is present worldwide, BLV genotyping studies are limited to certain areas, as shown in Table 4. Therefore, the accumulation of the full genome sequencing of BLV strains, assigned to different genotypes worldwide may define the genotype-dependent pathogenesis and association between genetic variability in each genotype and its infectivity, and differences in its functions in the future.

## Conclusion

BLV is the etiologic agent of EBL, which is the most common neoplastic disease in cattle. It infects cattle worldwide, thereby imposing a severe economic burden on the dairy cattle industry. In this review, we summarized currently available detailed information on BLV infection worldwide, and indicated that BLV has spread to most countries except for some countries which are completely free of BLV by successful BLV eradication. We also outlined at least ten different BLV genotypes circulating in various geographical locations worldwide and the distribution of these BLV genotypes worldwide. This should be useful information to those investigating BLV for the potential development of diagnostic methods and vaccines, and for reducing the incidence of BLV in herds.

## Abbreviations

AGID: Agar gel immunodiffusion
BI: Bayesian inference method
BLV: Bovine leukemia virus
EBL: Enzootic bovine leukosis
ELISA: Enzyme-linked immunosorbent assay
EPAHW: European Food Safety Authority panel on animal health and welfare
HTLV-I &-II: Human T-cell lymphotropic virus types I and II
LTR: Long terminal repeats
miRNA: microRNA
ML: Maximum likelihood method
NJ: Neighbor-joining method
PCR: Polymerase chain reaction
PHA: Passive hemagglutination assay
PL: Persistent lymphocytosis
qPCR: Quantitative PCR
RFLP: Restriction fragment length polymorphism
RIA: Radio immunoassay
STLV: Simian T-cell lymphotropic virus
